# Utilization of health care services among Medicare beneficiaries who visit federally qualified health centers

**DOI:** 10.1186/s12913-018-2847-x

**Published:** 2018-01-25

**Authors:** Tara A. Lavelle, Adam J. Rose, Justin W. Timbie, Claude M. Setodji, Suzanne G. Wensky, Katherine D. Giuriceo, Mark W. Friedberg, Rosalie Malsberger, Katherine L. Kahn

**Affiliations:** 1 RAND Corporation, 20 Park Plaza, Suite 920, Boston, MA 02116 USA; 2 Tufts University School of Medicine, Boston, MA USA; 3 Boston University School of Medicine, Boston, MA USA; 4 RAND Corporation, Arlington, VA USA; 5 RAND Corporation, Pittsburgh, PA USA; 6 Centers for Medicare & Medicaid Services, Baltimore, MD USA; 7 Harvard Medical School, Boston, MA USA; 8 RAND Corporation, Santa Monica, CA USA; 9 David Geffen School of Medicine at UCLA, Los Angeles, CA USA

**Keywords:** health care utilization, safety-net care, Medicare

## Abstract

**Background:**

Previous studies have disagreed on whether patients who receive primary care from federally qualified health centers (FQHCs) have different utilization patterns than patients who receive care elsewhere. Our objective was to compare patterns of healthcare utilization between Medicare beneficiaries who received primary care from FQHCs and Medicare beneficiaries who received primary care from another source.

**Methods:**

We compared characteristics and ambulatory, emergency department (ED), and inpatient utilization during 2013 between 130,637 Medicare beneficiaries who visited an FQHC for the majority of their primary care in 2013 (FQHC users) and a random sample of 1,000,000 Medicare fee-for-service (FFS) beneficiaries who did not visit an FQHC (FQHC non-users). We then created a propensity-matched sample of 130,569 FQHC users and 130,569 FQHC non-users to account for differences in observable patient characteristics between the two groups and repeated all comparisons.

**Results:**

Before matching, the two samples differed in terms of age (42% below age 65 for FQHC users vs. 16% among FQHC non-users, *p* < 0.001 for all comparisons), disability (52% vs. 24%), eligibility for Medicaid (56% vs. 21%), severe mental health disorders (17% vs. 10%), and substance abuse disorders (6% vs. 3%). FQHC users had fewer ambulatory visits to primary care or specialist providers (10.0 vs. 12.0 per year), more ED visits (1.2 vs. 0.8), and fewer hospitalizations (0.3 vs. 0.4). In the matched sample, FQHC users still had slightly lower utilization of ambulatory visits to primary care or specialist providers (10.0 vs. 11.2) and slightly higher utilization of ED visits (1.2 vs. 1.0), compared to FQHC users. Hospitalization rates between the two groups were similar (0.3 vs. 0.3).

**Conclusions:**

In this population of Medicare FFS beneficiaries, FQHC users had slightly lower utilization of ambulatory visits and slightly higher utilization of ED visits, compared to FQHC non-users, after accounting for differences in case mix. This study suggests that FQHC care and non-FQHC care are associated with broadly similar levels of healthcare utilization among Medicare FFS beneficiaries.

**Electronic supplementary material:**

The online version of this article (10.1186/s12913-018-2847-x) contains supplementary material, which is available to authorized users.

## Background

Federally qualified health centers (FQHCs) receive federal funding to provide comprehensive primary care in underserved communities. Three-quarters of the 20 million patients seen at FQHCs annually have incomes below the federal poverty level (FPL), and more than half are members of a racial or ethnic minority group [1]. In 2013, 35% of patients seen at FQHCs were uninsured, and another 49% had some type of public insurance including Medicaid and/or Medicare.^1^ The number of Medicare beneficiaries seen at FQHCs more than doubled between 2001 to 2011 (from 745,000 to nearly 1.6 million) [2].

Studies of FQHCs have consistently shown that they provide high quality primary care, but the results of research examining the overall healthcare utilization patterns of FQHC users have been mixed. Some studies have shown that FQHC users have more ambulatory visits, emergency department (ED) visits, and hospitalizations, but other studies show lower utilization [3–12]. Differences in these study outcomes relate in part to the segment of the population that was the focus of the study (e.g., younger patients, older patients, dual eligible patients), in part to the time period studied (since these studies span almost two decades), and in part to the extent that they controlled for important differences between FQHC users and FQHC non-users.

The purpose of this study was to examine the volume of ambulatory visits, ED visits, and hospitalizations among a sample of Medicare beneficiaries who visited an FQHC for a majority of their primary care visits in 2013, compared with a sample of beneficiaries who received regular primary care but did not visit an FQHC. These analyses used unadjusted direct comparisons between groups as well as comparisons between matched samples of beneficiaries that controlled for observable socio-demographic and clinical differences between groups. Our focus on Medicare beneficiaries is noteworthy, because while they represented just 8% of all beneficiaries who served by FQHCs in 2013 [2], they also are characterized by an especially high level of illness burden and medical need. We expected to show that FQHC users had higher levels of ED utilization before adjustment, but that adjustment for differences in case mix would greatly attenuate or eliminate this difference.

## Methods

### Sample

This study used 2013 Medicare fee-for-service (FFS) claim and enrollment files from a representative 20% sample of Medicare beneficiaries. Beneficiaries were included if, during all 12 months of 2013, they were at least 18 years of age, were eligible for both Parts A and B, and were not enrolled in Medicare Advantage. Our inclusion criteria also required beneficiaries to have at least three ambulatory visits to a primary care provider (PCP) during 2013. Physicians, nurse practitioners, and physician’s assistants in internal medicine, general practice, family medicine, obstetrics & gynecology, adult health, community health, family practice, primary care, women’s health, gerontology, and preventive medicine were classified as PCPs. PCPs were identified using unique National Provider Identifier (NPI) codes in the outpatient and physician claim files. These were linked to National Plan & Provider Enumeration System (NPPES) Provider Taxonomy codes to identify clinician type and specialty. PCP visits were defined as an evaluation and management visit in the Part B Medicare claims or an FQHC visit or rural health clinic (RHC) visit in the Part A Medicare claims.

**Fig. 1:**
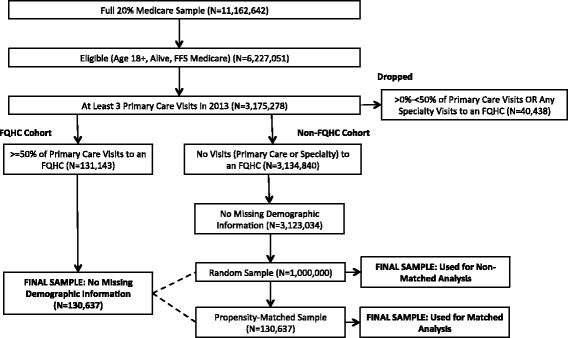
CONSORT flow diagram

Of those beneficiaries that met the inclusion criteria, we compared those who had at least half of their primary care visits at an FQHC (FQHC users) to a sample of beneficiaries who never visited an FQHC during 2013 (FQHC non-users). Those few beneficiaries who did visit an FQHC, either for primary or specialty care, but received more than half of their primary care elsewhere (1.3% of all beneficiaries), were excluded, as they could not be clearly attributed to FQHC vs. non-FQHC primary care. Among those beneficiaries who remained in the sample, 130,637 visited an FQHC for a majority of their primary care visits. Our comparison group consisted of a random sample of 1 million Medicare beneficiaries who did not visit an FQHC but received care from a PCP at a rural health clinic (RHC), private physician office, or other outpatient medical facility. The creation of our study cohort is depicted in Fig. 1 with a CONSORT diagram. Our study was approved by the RAND Corporation’s Human Subjects Protection Committee.

### Unadjusted analysis

We compared the unadjusted demographic and clinical characteristics of FQHC users and non-users using chi-square tests for categorical measures and t-tests for continuous measures. We adjusted standard errors to account for clustering at the site at which the beneficiary had the most PCP visits during the year. Characteristics derived from claims and enrollment files included age, race, gender, disability status, Medicaid eligibility, and prior institutionalization, defined as two or more stays at a skilled nursing facility in the previous 2 years. To measure burden of illness, we created indicators for clinical conditions based on the CMS Hierarchical Condition Categories (HCC) classification system [13]. This system assigns all ICD-9 diagnosis codes present on claims during a given year to 1 of 70 diagnostic HCC indicators [13]. We aggregated select HCC indicators to define the 15 most prevalent and clinically important comorbid condition classifications for FQHC users: cancer, cardiovascular disease, chronic heart failure, chronic lung disorders, diabetes, gastrointestinal (GI) disorders, human immunodeficiency virus (HIV), moderate or end-stage liver failure, neurological disorders, pancreatic disease, severe mental health disorders, stroke, substance abuse disorders, severe hematological disorders, and vascular disorders.

We used beneficiaries’ ZIP codes to derive the U.S. region and urban/rural classification of residence by using the rural-urban commuting area (RUCA) codes that classify U.S. census tracts based on population density, urbanization, and daily commuting [14]. We used six measures of neighborhood socioeconomic status (SES) generated from the 2009-2013 American Community Survey census data at the level of beneficiary ZIP Code Tabulation Areas (ZCTAs) mapped to beneficiary ZIP code, as proxy for individual SES [14]. These measures include median household income, percent of residents age 25 or older with less than a high school diploma, percent of male residents age 16 or older who are unemployed, percent of female-headed households with children, percent of households with public assistance income, and percent of individuals with annual income below the FPL [14].

We used descriptive statistics to compare the total volume of ambulatory visits to either a PCP or specialist, ED visits (which includes observation stays) with and without admission, and hospitalizations, between the FQHC user and non-user samples in 2013. Ambulatory visits were further divided into those that occurred at an FQHC and those that occurred in another setting, such as a private physician’s office or an RHC. Further, for each ED visit and hospitalization, we classified whether the primary diagnosis reported on the claim was one of nine chronic ambulatory care sensitive conditions (ACSCs): uncontrolled diabetes, diabetes with short-term complications (ketoacidosis, hyperosmolarity, coma), diabetes with long-term complications (renal, eye, neurological, or circulatory), lower-extremity amputation among patients with diabetes, chronic obstructive pulmonary disease (COPD) or asthma in older adults, asthma in younger adults, hypertension, congestive heart failure (CHF), or angina without procedure [15]. These are chronic conditions for which appropriate ambulatory care may reduce the need for services in an acute care hospital setting [15].

### Propensity-matched analysis

To examine the difference in health services utilization between FQHC users and non-users, controlling for observable differences in demographic and clinical characteristics, we created propensity score-matched groups. Propensity score matching is a technique intended to produce two groups that are as similar to each other as possible on a set of measured characteristics, and is a well-recognized approach to address confounding [16]. We created a score to characterize the propensity to receive primary care in an FQHC, including all the covariates described above. We then matched each FQHC user with a single FQHC non-user drawn from our pool of 1,000,000 using the “greedy matching” method, which is operationalized as follows. FQHC users are selected in a random order. As each FQHC user is selected, the algorithm looks among all available matches and chooses the one with the closest propensity score where closeness is measured in units of Euclidian distance. Matches that exceed the pre-specified caliper are not accepted; if no acceptable matches are available, the FQHC user is recorded as “not matched”. Once an FQHC non-user is matched to an FQHC user, that non-user is taken out of the pool of available matches. The process is repeated until all FQHC users have been matched or have been deemed as not matchable within the caliper [17]. We used a maximum caliper width of 0.2 standard deviations of the propensity score distribution (measured in log-odds), as recommended by Austin [18]. Because of this caliper, 68 FQHC users could not be matched to a control and were therefore excluded from the PS-matched analyses. We repeated our comparison of rates of ambulatory visits, ED visits, and hospitalizations using the matched samples. We also repeated the analyses using an alternative (narrower) caliper of 0.1 standard deviations, but the results were similar and are not reported here.

### Sensitivity analyses – Methods

In addition to our main analyses, we conducted six sensitivity analyses to test the strength of our findings. In the first sensitivity analysis, we restricted the sample to those beneficiaries who had met the eligibility criteria in the year prior (2012) as well as the study year (2013) to ensure analysis of beneficiaries with a more consistent source of care and whose primary care providers may therefore have had more opportunity to affect utilization. FQHC users were defined for this sensitivity analysis as those for whom the majority of primary care visits in both years were to an FQHC. The comparison group was drawn from those beneficiaries who had at least 3 primary care visits in both years but did not visit an FQHC in either year. We matched FQHC users to FQHC non-users and repeated all of our analyses.

In the second sensitivity analysis, we stratified the sample to those age 65 and above and those age 64 and below. We reasoned that the younger Medicare beneficiaries might be systematically different from older ones, because receiving Medicare coverage below age 65 specifically requires disability or renal failure. We matched each cohort of FQHC users (younger, older) with FQHC non-users from the same age group and repeated all analyses.

In the third sensitivity analysis, we addressed concerns about the presence of RHCs in the comparison group. For the primary analysis, we included RHCs in the comparison group to provide rural beneficiaries as a potential match for the many rural FQHC Medicare beneficiaries. However, we were concerned that similarities in regulatory requirements between FQHCs and RHCs might obscure differences we would observe between FQHC users and FQHC non-users. We therefore created an alternative comparison group, excluding any beneficiary who had visited an RHC for any reason. We again selected 1 million random beneficiaries from the available pool, matched 130,637 of them, one to each FQHC user, and repeated all analyses.

In the fourth sensitivity analysis, we examined a possible relationship between access to specialty care and differences in utilization between FQHC users and non-users. We restricted the sample of FQHC users, and the potential pool of FQHC non-users, to those who had at least one specialty visit in 2013. We repeated the propensity score match and repeated all analyses. In the fifth sensitivity analysis, we restricted the sample to beneficiaries who had at least three specialty visits in 2013, rather than one. By limiting these analyses to beneficiaries who had seen a specialist, we examined whether there was any attenuation of differences among beneficiaries who presumably had at least some access to specialty care.

In the sixth sensitivity analysis, we repeated our main utilization comparison between FQHC users and non-users, without propensity matching, but with multivariate control for age and burden of illness, defined as the individual conditions that are part of the HCC score, plus the presence or absence of end-stage renal disease. By comparing these results with the fully propensity matched results, we aimed to provide insight into how much of the apparent difference between FQHCs and other models of care may actually be due to measureable socioeconomic inputs, which are controlled for in the propensity matched model but not the partially-adjusted model. All analyses were conducted with SAS, version 9.4 (SAS Corporation, Cary, NC).

## Results

### Unadjusted comparison of demographic and clinical characteristics

We observed important differences between the samples of FQHC users and non-users in the unadjusted analysis (Table 1). FQHC users were significantly more likely than FQHC non-users to be under 65 years old (42% vs. 16%, *p* < 0.001 for this and all other comparisons in this section), non-White (32% vs. 15%), disabled (52% vs. 24%), and Medicaid-eligible (56% vs. 21%). FQHC users were more likely to have certain chronic conditions compared with FQHC non-users, including diabetes (40% vs. 37%), severe mental health disorders (18% vs. 11%), and substance abuse disorders (6% vs. 3%). However, FQHC users were less likely to have been diagnosed with cancer (11% vs. 17%), cardiovascular disease (16% vs. 25%), or another vascular disorder (15% vs. 24%). Many of these differences remained even after stratifying the FQHC user versus non-user comparisons by age (over and under 65 years; results not shown).

**Table 1:** Characteristics of Medicare Beneficiaries with a Majority of Primary Care Clinician Visits to an FQHC in 2013 Compared with Medicare Beneficiaries Who Received Primary Care Elsewhere

Characteristic	FQHC Users (*n* = 130,569)	Unmatched FQHC Non-Users (*n* = 1,000,000)	Matched FQHC Non-Users (*n* = 130,569)
Demographics			
Age			
18-64	42.3%	16.1%†	42.7%
65-74	36.1%	40.9%†	35.7%
75-84	16.2%	29.1%†	16.3%
85+	5.4%	13.9%†	5.3%
Race/Ethnicity			
White	67.7%	85.2%†	67.6%
Black	18.8%	9.1%†	19.8%
Hispanic	7.1%	1.7%†	6.0%
Asian	2.7%	1.7%†	2.9%
Other/Unknown	3.7%	2.3%†	3.7%
Gender			
Male	41.4%	38.8%†	40.8%*
Female	58.6%	61.2%†	59.2%
Disabled	52.2%	23.9%†	52.9%
End-Stage Renal Disease Status	0.8%	0.6%†	0.9%
Previously Institutionalized	3.3%	8.1%†	3.3%
Medicaid-Eligible	56.5%	21.0%†	56.8%
Region			
Northeast	19.7%	18.2%†	19.0%
Midwest	18.1%	23.6%†	18.7%
South	34.2%	42.4%†	36.5%
West	28.0%	15.8%†	25.7%
Urban/Rural Status			
Urban	72.8%	84.6%†	72.6%
Rural/Isolated	27.2%	15.4%†	27.4%
Neighborhood Socioeconomic Status (SES)			
Median Annual Household Income, Mean	$44,565	$55,812†	$44,301
Percent of Residents with Less than High School Diploma (Age 25+), Mean	18.4%	13.4%†	18.5%
Percent of Male Residents Unemployed (Age 16+), Mean	12.4%	10.3%†	12.5%
Percent of Female-Headed Households with Children, Mean	12.8%	10.6%†	13.0%
Percent of Households with Public Assistance Income, Mean	3.8%	2.6%†	3.8%
Percent of Individuals with Annual Income Below Federal Poverty Level, Mean	20.1%	14.8%†	20.3%
Comorbid Conditions			
Cancer	10.9%	17.0%†	10.7%
Cardiovascular Disease	16.1%	24.8%†	16.2%
Chronic Heart Failure	16.8%	21.3%†	17.1%
Chronic Lung Disease	19.4%	19.3%	19.9%*
Diabetes	40.5%	37.2%†	41.2%*
Gastrointestinal Disorders	3.0%	4.2%†	3.1%
HIV	1.3%	0.3%†	1.2%
Moderate or End-Stage Liver Disease	3.2%	1.8%†	3.2%
Neurological Disorders	17.9%	19.0%†	18.3%*
Pancreatic Disease	2.0%	2.3%†	2.0%
Severe Mental Health Disorders	17.5%	10.6%†	17.5%
Stroke	5.6%	7.6%†	5.7%
Substance Abuse Disorders	5.6%	2.7%†	5.5%
Severe Hematological Disorders	0.5%	0.8%†	0.5%
Vascular Disorders	14.8%	23.5%†	15.2%*
Total Number of Comorbid Conditions, Mean	1.8	1.9†	1.8*

The unmatched analysis compares FQHC users and all 1,000,000 FQHC non-users. The matched analysis compares FQHC users and a subset of all 1,000,000 non-users after a 1:1 propensity match between FQHC users and FQHC non-users

*p < 0.05; †*p* < 0.001

*FQHC* Federally Qualified Health Center

We also observed important differences between groups regarding living situation (Table 1). FQHC users were more likely to live in a rural area (27% vs. 15%). FQHC users also lived in ZIP codes with a lower median household income ($44,560 vs. $55,812), higher mean percent of residents with less than a high school diploma (18% vs. 13%), and higher mean percent of residents with an annual income below the FPL (20% vs. 15%).

### Unadjusted comparison of utilization

FQHC users had fewer ambulatory visits during the year (10.0; see Table 2) compared with FQHC non-users (12.0; *p* < 0.001). Much of the difference in ambulatory visits was driven by fewer ambulatory specialty care visits among FQHC users vs. non-users (3.8 vs. 5.3, *p* < 0.001). Among FQHC users, 77.3% had an ambulatory visit in a non-FQHC setting (including physician office, RHC, or other setting) during 2013. FQHC users had a considerably higher rate of ED visits. FQHC users had a mean of 1.2 ED visits per year whereas FQHC non-users had 0.8 visits per year (*p* < 0.001). Forty-four percent of FQHC users had at least one ED visit during the year, compared with 39% among FQHC non-users (*p* < 0.001). Overall the majority of ED visits in both groups did not result in admission. Most ED visits were not due to an ACSC, although the rate of visits for ACSCs was somewhat higher among the FQHC users than the FQHC non-users (5.9% vs. 4.6% with at least one ED visit for an ACSC during the year, *p* < 0.001).

**Table 2:** Comparison of Healthcare Utilization Between Medicare Beneficiaries with a Majority of Primary Care Clinician Visits to an FQHC in 2013 and Medicare Beneficiaries Who Received Primary Care Elsewhere

	FQHC Users (*n* = 130,569)		Unmatched FQHC Non-Users (*n* = 1,000,000)		Matched FQHC Non-Users (*n* = 130,569)	
	Mean number of visits	Percent of beneficiaries with at least one	Mean number of visits	Percent of beneficiaries with at least one	Mean number of visits	Percent of beneficiaries with at least one
Ambulatory Visits						
Any ambulatory visit	10.0	100.0%	12.0†	100.0%	11.2†	100.0%
Primary care	6.2	100.0%	6.7†	100.0%	6.8†	100.0%
Specialty care	3.8	73.7%	5.3†	83.8%†	4.5†	78.1%†
At an FQHC	5.8	100.0%	0.0†	0.0%†	0.0†	0.0%†
Primary care	5.5	100.0%	0.0†	0.0%†	0.0†	0.0%†
Specialty care	0.3	9.8%	0.0†	0.0%†	0.0†	0.0%†
At site other than FQHC	4.2	77.3%	12.0†	100.0%†	11.2†	100.0%†
Primary care	0.7	33.7%	6.7†	100.0%†	6.8†	100.0%†
Specialty care	3.5	70.9%	5.3†	83.8%†	4.5†	78.1%†
ED visits						
Any ED visit	1.2	44.5%	0.8†	39.3%†	1.0†	41.5%†
Chronic ACSC	0.1	5.9%	0.1†	4.6%†	0.1†	4.8%†
Other	1.1	42.7%	0.8†	37.8%t	0.9†	40.0%†
With admission only	0.2	13.3%	0.2†	15.8%†	0.2*	12.7%†
Chronic ACSC	0.0	2.2%	0.0	2.3%	0.0*	2.0%*
Other	0.2	11.9%	0.2†	14.6%†	0.2*	11.4%*
Without admission only	1.0	39.8%	0.6†	31.9%†	0.8†	36.4%†
Chronic ACSC	0.1	4.3%	0.0†	2.7%†	0.0†	3.3%†
Other	0.9	38.3%	0.6†	30.8%†	0.8†	35.2%†
Hospitalizations						
Any hospitalization	0.3	19.1%	0.4†	23.0%†	0.3	18.8%
Chronic ACSC	0.0	2.9%	0.0	2.9%	0.0	2.6%*
Other	0.3	17.6%	0.3†	21.7%†	0.3	17.5%

The unmatched analysis compares FQHC users and all 1,000,000 FQHC non-users. The matched analysis compares FQHC users and a subset of all 1,000,000 non-users after a 1:1 propensity match between FQHC users and FQHC non-users

*p < 0.05; †*p* < 0.001

*FQHC* Federally Qualified Health Center, *ACSC* Ambulatory Care Sensitive Condition, *ED* Emergency Department

Differences between FQHC users and non-users with regard to inpatient care were relatively small, although on some measures, FQHC users had less inpatient utilization. FQHC users had a mean of 0.3 hospitalizations per year whereas FQHC non-users had 0.4 hospitalizations per year (*p* < 0.001). Nineteen percent of FQHC users were hospitalized at least once in 2013, compared with 23% of FQHC non-users (*p* < 0.001).

### Matched comparison of utilization

Propensity matching created a sample of 130,569 FQHC users and 130,569 matched FQHC non-users (Table 1). After matching, differences between samples on demographic and clinical characteristics were negligible, although some comparisons still achieved statistical significance due to the large sample size. Comparing the matched samples, FQHC users still used somewhat less ambulatory care than FQHC non-users (10.0 vs. 11.2, *p* < 0.001; Table 2). FQHC users still had fewer ambulatory visits for specialty care, although the difference was attenuated from the unadjusted analysis (3.8 vs. 4.5, p < 0.001). The mean number of ED visits was more similar between groups than in the unmatched comparison (1.2 vs. 1.0, p < 0.001). The mean number of hospitalizations remained similar for both groups (0.3 hospitalizations per year, *p* = 0.12).

### Sensitivity analyses

The first sensitivity analysis (Additional file 1: Tables S1 and S2) used a sample of beneficiaries who had met the eligibility criteria both in the year prior (2012) as well as the study year (2013). Application of this requirement for FQHC visits in two consecutive years eliminated 50,718 of 130,637 (39%) of the FQHC users. We created a propensity-matched sample of FQHC non-users who also had met the eligibility criteria in both 2012 and 2013. The comparison of utilization was essentially unchanged from the main analysis.

In the second sensitivity analysis (Additional file 1: Tables S3-S6), we stratified the sample to those age 65 and above and those age 64 and below. As would be expected, there were differences between the younger and older cohorts on baseline characteristics. Utilization of ambulatory visits and especially ED visits was considerably higher in the under-65 age group than in the older beneficiaries. However, the extent of differences between FQHC users and FQHC non-users within each age stratum was similar to what was seen in the main analysis, and thus our main results were essentially unchanged.

In the third sensitivity analysis (Additional file 1: Tables S7 and S8), we removed all beneficiaries from the non-FQHC group who had visited a rural health clinic for any reason during 2013. This eliminated 128,056 (4.1%) of the 3,123,034 potential FQHC non-users. We randomly selected 1,000,000 of these FQHC non-users and created a propensity-matched sample with the 130,637 FQHC users, as we had in the main analysis. The results are almost indistinguishable from those of the main analysis.

In the fourth and fifth sensitivity analyses (Additional file 1: Tables S9-S12), we restricted FQHC users and non-users to those who had at least one specialty visit, and then at least three specialty visits, in 2013. The group with at least one specialty visit constituted 96,188 FQHC users and the same number of matched controls, while the requirement for at least three specialty visits reduced this sample to 61,908 FQHC users. Restriction of the sample did result in an even tighter match of the two samples on measured characteristics (see Additional file 1: Tables S9 and S11), such that the match for those with at least 3 specialty visits did not contain any statistically significant differences, which is noteworthy given the large size of the dataset. Predictably, beneficiaries had a higher burden of illness as we required them to have more specialist visits, and their utilization of all health services was increased. The small relative differences in utilization between FQHC users and non-users seen in the main analysis (slightly fewer ambulatory visits, slightly more ED visits) remained essentially unchanged. In short, this analysis did not particularly support the hypothesis that differential access to specialty care is driving utilization differences between FQHC users and non-users, in that removing such access as a consideration did not meaningfully impact relative differences in utilization.

Finally, in the sixth sensitivity analysis (Additional file 1: Table S13), we examined differences in utilization between FQHC users and non-users without propensity matching, but with multivariate control for age and burden of illness. The adjusted results were extremely similar to our main analysis (manuscript, Table 2), with an approximately 20% higher utilization of ED visits by FQHC users, relative to non-users. This result implies that the main drivers of increased ED visits among FQHC users are in fact age and burden of illness, whereas the additional attenuation from controlling for other measured variables (such as area-level socioeconomic status) is slight.

## Discussion

We compared beneficiary characteristics and the utilization of ambulatory, ED, and inpatient hospital care between Medicare beneficiaries who received a majority of their primary care at an FQHC during 2013 and beneficiaries who received primary care elsewhere. We found that these groups were extremely different, in terms of socio-demographic characteristics and burden of illness. In the unadjusted analysis, FQHC users had fewer ambulatory visits, and were hospitalized somewhat less but they visited the ED much more frequently (about 50% more visits) compared to FQHC non-users. However, after using propensity score matching to create a group of FQHC non-users similar to the FQHC users based on observable characteristics, the difference in utilization patterns was attenuated. This was most observable in terms of ED utilization, as the 50% difference in rates between groups was reduced to 20%. Sensitivity analyses demonstrated that this result was robust to various methodological choices.

Previous studies that have examined health care utilization among FQHC users have produced mixed results. Most of the previous studies adjusted for patient-level characteristics, either using propensity score matching, as we did here, or multivariable analysis. These previous analyses had mixed findings, with some showing lower rates of ambulatory, ED, or hospital utilization while others showing higher rates, although the effect sizes tend to be small [3–12]. To some extent, these variable results may relate to differences in methods, such as which subset of the population was examined or how adjustment was accomplished.

Most recently, Chang et al. focused on Medicare beneficiaries over age 65, and characterized FQHC users as “predominant users” (for whom the majority of visits were to an FQHC) and “non-predominant users” (for whom less than half of their visits were to an FQHC). They found that predominant users had lower utilization of both ambulatory and inpatient care, while non-predominant users had higher utilization of both [11]. However, Chang et al. did not specifically examine ED visits, so our report provides new findings in that regard. Wright et al. found that Black and Hispanic dual-eligibles (those covered by both Medicaid and Medicare) had lower rates of hospitalizations and ED visits for ambulatory care sensitive conditions (ACSCs) if they received care at an FQHC than if they did not [10]. Potter et al. focused on dual-eligibles under age 65, and found that minority patients had slightly more hospitalizations and ED visits for ACSCs if they used an FQHC, while White patients had slightly fewer if they used an FQHC [12]. While these three recent studies examined different subsets of FQHC users, and reached somewhat different conclusions, they do have in common that they all found small effect sizes, on the order of a 20% difference in utilization between groups, or less [10–12].

The results of our study are generally consistent with these previous findings. We found that, in the unadjusted analyses, FQHC users had fewer ambulatory visits and hospitalizations, but they visited the ED more frequently than FQHC non-users. After adjusting for case mix, this difference was considerably attenuated, particularly with regards to the ER visits, but not eliminated. Like the other studies, the differences in utilization that we found between FQHC users and FQHC non-users are small: less than a 20% relative difference in ambulatory visits, and less than a 20% relative difference in ED visits, after adjustment via propensity score matching. While these differences are small in relative terms, they could be a relevant consideration for policy makers. In 2013, FQHCs served about 1.7 million Medicare beneficiaries [2]. The results of our propensity-matched analysis imply that, after accounting for differences in case mix, these 1.7 million FQHC users would be expected to use 1.3 fewer ambulatory visits per beneficiary per year, or approximately 2.2 million fewer ambulatory visits, than if they had received their primary care elsewhere. On the other hand, they would be expected to use 0.2 additional ED visits per beneficiary per year, or approximately 340,000 more ED visits. The policy relevance of these small differences should be considered in the context of the unique role of FQHCs in providing services to some of our most vulnerable patients. While FQHCs are not the only source of care for underserved populations, they are uniquely committed to this population as part of their mission statement and charter [2], and thus form an essential part of our national approach to providing such care.

In our study, we found that differences in utilization between FQHC users and FQHC non-users were greatly attenuated, but not completely eliminated, by controlling for confounding through propensity matching. It is possible that the small residual differences in healthcare utilization that persisted after adjustment are attributable to residual unmeasured differences in unmeasured clinical characteristics between the matched groups. Another possibility is that the residual difference represents a true difference in ambulatory visit and ED utilization patterns between FQHC users and non-users that otherwise look the same in terms of socio-demographic and clinical characteristics and had a similar likelihood of receiving care at an FQHC. This in turn could reflect subtle differences in the way that care is provided between FQHCs and other primary care clinics.

One key limitation of this study is that we were not able to completely account or adjust for continuity of care as the available data were not sufficient to characterize continuity of care. An extensive body of research has shown the key importance of continuity of care on predicting health care utilization and outcomes [19]. If our FQHC user group had higher continuity of care versus our comparison group, our associations between the FQHC use and health care utilization may reflect unmeasured confounding with continuity of care. Also, our focus on Medicare beneficiaries is both a strength and a limitation for this study. It is a strength because it enabled us to focus on a sub-population that has extremely high medical need and illness burden. However, because Medicare beneficiaries only account for 8% of all FQHC users [2], our results may not be completely generalizable to other populations.

## Conclusions

In conclusion, studying a population of Medicare FFS beneficiaries, we found that FQHC users had fewer ambulatory visits and more visits to the ED compared to a matched comparison group of beneficiaries who receive primary care from another source, but the magnitude of these differences was small in relative terms. This study suggests that FQHC care and non-FQHC care are associated with broadly similar levels of healthcare utilization among Medicare FFS beneficiaries.

## Additional file

Additional file 1: Sensitivity Analyses. (DOCX 77 kb)

## Abbreviations

ACSC: Ambulatory Care Sensitive Conditions
CHF: Congestive Heart Failure
CMS: Centers for Medicare and Medicaid Services
COPD: Chronic Obstructive Pulmonary Disease
ED: Emergency Department
FFS: Fee For Service
FPL: Federal Poverty Level
FQHC: Federally Qualified Health Center
GI: Gastrointestinal
HCC: Hierarchical Condition Comorbidity
HIV: Human Immunodeficiency Virus
ICD-9: International Classification of Diseases Clinical Modification 9th Revision
NPI: National Provider Identifier
NPPES: National Plan and Provider Enumeration System
PCP: Primary Care Provider
RHC: Rural Health Center
RUCA: Rural-Urban Commuting Area
SES: Socioeconomic Status
ZTCA: Zip Code Tabulation Area
