# Supplementary material for: Utilization of health care services among Medicare beneficiaries who visit federally qualified health centers
Source: BMC Health Serv Res. 2018 Jan 25;18:41. doi: 10.1186/s12913-018-2847-x (PMC5785864; doi:10.1186/s12913-018-2847-x)
Supplement: Additional file 1: — Sensitivity Analyses. (DOCX 77 kb) [file 12913_2018_2847_MOESM1_ESM.docx]

Online Appendix: Sensitivity Analyses

Appendix Table 1: Comparison of baseline characteristics between 79,906 Medicare beneficiaries who received primary care at Federally Qualified Health Centers both in 2012 and 2013, and 79,906 propensity-matched Medicare beneficiaries who received primary care elsewhere. This analysis differs from the main analysis in that all beneficiaries met the entry criteria for two years in a row (2012 and 2013).

| **Characteristic** | **FQHC Users**  **(n=79,906)** | **FQHC Non-Users**  **(n=79,906)** |
| --- | --- | --- |
| **Demographics** |  |  |
| Age |  |  |
| 18-64 | 40.2% | 40.6% |
| 65-74 | 36.0% | 35.6% |
| 75-84 | 17.9% | 18.0% |
| >=85 | 6.0% | 5.8% |
| Race/Ethnicity |  |  |
| White | 67.1% | 66.8% |
| Black | 18.6% | 19.8% |
| Hispanic | 7.5% | 6.5% |
| Asian | 3.0% | 3.2% |
| Other/ Unknown | 3.8% | 3.6% |
| Gender* |  |  |
| Male | 39.9% | 39.3% |
| Female | 60.1% | 60.7% |
| Disabled | 51.7% | 52.5% |
| End-Stage Renal Disease (ESRD) status | 0.8% | 0.8% |
| Previously Institutionalized | 3.2% | 3.2% |
| Medicaid-eligible | 58.0% | 58.6% |
| Region |  |  |
| Northeast | 20.2% | 19.5% |
| Midwest | 17.3% | 17.8% |
| South | 34.0% | 36.1% |
| West | 28.5% | 26.6% |
| Urban/Rural status, % |  |  |
| Urban | 71.7% | 72.2% |
| Rural/ Isolated | 28.3% | 27.8% |
| **Neighborhood socioeconomic status (SES)** |  |  |
| Median annual household income, mean | $44,302 | $44,023 |
| Percent of residents with less than high school diploma (age 25+), mean | 18.8% | 18.8% |
| Percent of male residents who are unemployed (age 16+), mean | 12.5% | 12.7% |
| Percent of female-headed households with children, mean* | 12.7% | 13.1% |
| Percent of households with public assistance income, mean | 3.9% | 3.9% |
| Percent of individuals with annual income below the FPL, mean | 20.2% | 20.5% |
| **Comorbid Conditions** |  |  |
| Cancer | 11.4% | 11.2% |
| Cardiovascular disease | 17.2% | 17.4% |
| Chronic heart failure | 17.8% | 18.1% |
| Chronic lung disorders | 20.8% | 21.3% |
| Diabetes | 44.3% | 44.9% |
| Gastrointestinal disorders | 3.1% | 3.2% |
| HIV | 1.3% | 1.1% |
| Moderate or end-stage liver disease | 3.3% | 3.1% |
| Neurological disorders | 18.2% | 18.6% |
| Pancreatic disease | 2.1% | 2.1% |
| Severe mental health disorders | 16.8% | 17.0% |
| Stroke | 5.6% | 5.6% |
| Substance abuse disorders | 5.0% | 4.8% |
| Severe hematological disorders | 0.5% | 0.5% |
| Vascular disorders | 15.8% | 16.0% |
| **Total number of co-morbid conditions, mean** | **1.8** | **1.8** |
| *p<0.05 for difference between FQHC users and FQHC Non-Users. | | |

Appendix Table 2: Comparison of utilization between 79,906 Medicare beneficiaries who received primary care at Federally Qualified Health Centers both in 2012 and 2013, and 79,906 propensity-matched Medicare beneficiaries who received primary care elsewhere. This analysis differs from the main analysis in that all beneficiaries met the entry criteria for two years in a row (2012 and 2013).

|  | **FQHC Users**  **(n=79,906)** | | | **Matched FQHC Non-Users**  **(n=79,906)** | |  |
| --- | --- | --- | --- | --- | --- | --- |
|  | |  |  |  |  |  |
|  | | **Mean number of visits** | **Percent of benes with at least one** | **Mean number of visits** | **Percent of benes with at**  **least one** |  |
| **Ambulatory visits** | |  |  |  |  |  |
| Any ambulatory visit | | 10.7† | 100.0% | 12.0 | 100.0% |  |
| Primary care | | 6.6† | 100.0% | 7.3 | 100.0% |  |
| Specialty care | | 4.1† | 75.8%† | 4.7 | 79.1% |  |
| At an FQHC | | 6.3† | 100.0%† | 0.0 | 0.0% |  |
| Primary care | | 6.0† | 100.0%† | 0.0 | 0.0% |  |
| Specialty care | | 0.3† | 10.5%† | 0.0 | 0.0% |  |
| At site other than FQHC | | 4.4† | 77.8%† | 12.0 | 100.0% |  |
| Primary care | | 0.6† | 30.0%† | 7.3 | 100.0% |  |
| Specialty care | | 3.8† | 73.0%† | 4.7 | 79.1% |  |
| **ED visits** | |  |  |  |  |  |
| Any ED visit | | 1.1† | 44.1%† | 1.0 | 41.5% |  |
| Chronic ACSC | | 0.1† | 6.0%† | 0.1 | 4.9% |  |
| Other | | 1.0† | 42.3%† | 0.9 | 40.0% |  |
| With admission only | | 0.2* | 13.0%* | 0.2 | 12.4% |  |
| Chronic ACSC | | 0.0† | 2.3%† | 0.0 | 2.0% |  |
| Other | | 0.2 | 11.6% | 0.2 | 11.2% |  |
| Without admission only | | 0.9† | 39.5%† | 0.8 | 36.6% |  |
| Chronic ACSC | | 0.1† | 4.2%† | 0.0 | 3.4% |  |
| Other | | 0.9† | 38.1%† | 0.8 | 35.4% |  |
| **Hospitalizations** | |  |  |  |  |  |
| Any hospitalization | | 0.3* | 18.9%* | 0.3 | 18.3% |  |
| Chronic ACSC | | 0.0* | 2.9%* | 0.0 | 2.7% |  |
| Other | | 0.3* | 17.4%* | 0.3 | 16.9% |  |
| For comparison with FQHC Non-Users: *p<0.05; †p<0.001; otherwise not statistically significant.  FQHC: Federally-qualified health center; ACSC: Ambulatory Care Sensitive Condition; ED: Emergency Department; E&M: Evaluation and Management. | | | | | | |
|  | | | | | | |

Appendix Table 3: Comparison of baseline characteristics between 55,342 Medicare beneficiaries under age 65 who received primary care at Federally Qualified Health Centers in 2013, and 55,342 propensity-matched Medicare beneficiaries under age 65 who received primary care elsewhere.

| **Characteristic** | **FQHC Users**  **(n=55,342)** | **FQHC Non-Users**  **(n=55,342)** |
| --- | --- | --- |
| **Demographics** |  |  |
| Age |  |  |
| 18-64 | 100% | 100% |
| 65-74 | -- | -- |
| 75-84 | -- | -- |
| >=85 | -- | -- |
| Race/Ethnicity |  |  |
| White | 64.8% | 64.3% |
| Black | 23.2% | 24.2% |
| Hispanic | 7.2% | 7.0% |
| Asian | 1.4% | 1.3% |
| Other/ Unknown | 3.4% | 3.2% |
| Gender |  |  |
| Male | 46.4% | 45.8% |
| Female | 53.6% | 54.2% |
| Disabled | 98.7%* | 98.9% |
| End-Stage Renal Disease (ESRD) status | 1.8% | 1.6% |
| Previously Institutionalized | 1.9%* | 1.7% |
| Medicaid-eligible | 74.3% | 74.2% |
| Region |  |  |
| Northeast | 20.3% | 19.7% |
| Midwest | 20.5% | 21.0% |
| South | 32.7% | 33.9% |
| West | 26.5% | 25.4% |
| Urban/Rural status, % |  |  |
| Urban | 78.6% | 79.3% |
| Rural/ Isolated | 21.4% | 20.7% |
| **Neighborhood socioeconomic status (SES)** |  |  |
| Median annual household income, mean | $44,348 | $44,298 |
| Percent of residents with less than high school diploma (age 25+), mean | 18.1% | 18.2% |
| Percent of male residents who are unemployed (age 16+), mean | 12.8% | 12.9% |
| Percent of female-headed households with children, mean | 13.7% | 14.0% |
| Percent of households with public assistance income, mean | 4.0% | 4.1% |
| Percent of individuals with annual income below the FPL, mean | 20.7% | 20.8% |
| **Comorbid Conditions** |  |  |
| Cancer | 7.9%* | 7.3% |
| Cardiovascular disease | 10.5%* | 9.9% |
| Chronic heart failure | 14.6%* | 13.7% |
| Chronic lung disorders | 19.4%* | 18.7% |
| Diabetes | 38.0% | 37.7% |
| Gastrointestinal disorders | 3.3%* | 3.0% |
| HIV | 2.6% | 2.4% |
| Moderate or end-stage liver disease | 5.2%* | 4.8% |
| Neurological disorders | 23.8%* | 23.1% |
| Pancreatic disease | 2.5% | 2.4% |
| Severe mental health disorders | 32.6% | 32.2% |
| Stroke | 5.3% | 5.0% |
| Substance abuse disorders | 10.6%* | 9.9% |
| Severe hematological disorders | 0.4% | 0.4% |
| Vascular disorders | 11.0%* | 10.3% |
| **Total number of co-morbid conditions, mean** | **1.9*** | **1.8** |
| *p<0.05 for difference between FQHC users and FQHC Non-Users. | | |

Appendix Table 4: Comparison of utilization between 55,342 Medicare beneficiaries under age 65 who received primary care at Federally Qualified Health Centers in 2013, and 55,342 propensity-matched Medicare beneficiaries under age 65 who received primary care elsewhere.

|  | | **FQHC Users**  **(n=55,342)** | | **Matched FQHC Non-Users**  **(n=55,342)** | |  |
| --- | --- | --- | --- | --- | --- | --- |
|  |  | |  |  |  |  |
|  | **Mean number of visits** | | **Percent of benes with at least one** | **Mean number of visits** | **Percent of benes with at**  **least one** |  |
| **Ambulatory visits** |  | |  |  |  |  |
| Any ambulatory visit | 10.6† | | 100.0% | 11.7 | 100.0% |  |
| Primary care | 6.4† | | 100.0% | 6.9 | 100.0% |  |
| Specialty care | 4.2† | | 74.1%† | 4.8 | 77.3% |  |
| At an FQHC | 6.0† | | 100.0%† | 0.0 | 0.0% |  |
| Primary care | 5.6† | | 100.0%† | 0.0 | 0.0% |  |
| Specialty care | 0.4† | | 11.7%† | 0.0 | 0.0% |  |
| At site other than FQHC | 4.5† | | 77.7%† | 11.7 | 100.0% |  |
| Primary care | 0.8† | | 35.8%† | 6.9 | 100.0% |  |
| Specialty care | 3.8† | | 70.8%† | 4.8 | 77.3% |  |
| **ED visits** |  | |  |  |  |  |
| Any ED visit | 1.6† | | 52.4%† | 1.4 | 48.1% |  |
| Chronic ACSC | 0.1† | | 6.6%† | 0.1 | 5.0% |  |
| Other | 1.5† | | 50.9%† | 1.3 | 46.9% |  |
| With admission only | 0.2† | | 14.2%† | 0.2 | 12.7% |  |
| Chronic ACSC | 0.0* | | 2.4%† | 0.0 | 1.9% |  |
| Other | 0.2† | | 12.8%† | 0.2 | 11.6% |  |
| Without admission only | 1.4† | | 48.7%† | 1.2 | 44.2% |  |
| Chronic ACSC | 0.1† | | 4.9%† | 0.1 | 3.6% |  |
| Other | 1.3† | | 47.4%† | 1.1 | 43.2% |  |
| **Hospitalizations** |  | |  |  |  |  |
| Any hospitalization | 0.3† | | 19.3%† | 0.3 | 17.9% |  |
| Chronic ACSC | 0.0* | | 3.0%† | 0.0 | 2.4% |  |
| Other | 0.3† | | 17.8%† | 0.3 | 16.7% |  |
| For comparison with FQHC Non-Users: *p<0.05; †p<0.001; otherwise not statistically significant.  FQHC: Federally-qualified health center; ACSC: Ambulatory Care Sensitive Condition; ED: Emergency Department; E&M: Evaluation and Management. | | | | | | |
|  | | | | | | |

Appendix Table 5: Comparison of baseline characteristics between 75,246 Medicare beneficiaries age 65 or older who received primary care at Federally Qualified Health Centers in 2013, and 75,246 propensity-matched Medicare beneficiaries age 65 or older who received primary care elsewhere.

| **Characteristic** | **FQHC Users**  **(n=75,246)** | **FQHC Non-Users**  **(n=75,246)** |
| --- | --- | --- |
| **Demographics** |  |  |
| Age* |  |  |
| 18-64 | -- | -- |
| 65-74 | 62.6% | 61.5% |
| 75-84 | 28.1% | 28.8% |
| >=85 | 9.4% | 9.6% |
| Race/Ethnicity |  |  |
| White | 69.8% | 69.5% |
| Black | 15.5% | 16.6% |
| Hispanic | 7.0% | 6.1% |
| Asian | 3.7% | 4.0% |
| Other/ Unknown | 3.9% | 3.8% |
| Gender |  |  |
| Male | 37.8% | 37.2% |
| Female | 62.2% | 62.8% |
| Disabled | 18.0% | 18.2% |
| End-Stage Renal Disease (ESRD) status | 0.2% | 0.2% |
| Previously Institutionalized | 4.3% | 4.4% |
| Medicaid-eligible | 43.4% | 43.5% |
| Region |  |  |
| Northeast | 19.2% | 18.4% |
| Midwest | 16.3% | 16.7% |
| South | 35.3% | 37.2% |
| West | 29.1% | 27.6% |
| Urban/Rural status, % |  |  |
| Urban | 68.6% | 68.6% |
| Rural/ Isolated | 31.4% | 31.4% |
| **Neighborhood socioeconomic status (SES)** |  |  |
| Median annual household income, mean | $44,722 | $44,538 |
| Percent of residents with less than high school diploma (age 25+), mean | 18.6% | 18.7% |
| Percent of male residents who are unemployed (age 16+), mean | 12.1% | 12.3% |
| Percent of female-headed households with children, mean | 12.1%* | 12.4% |
| Percent of households with public assistance income, mean | 3.7% | 3.7% |
| Percent of individuals with annual income below the FPL, mean | 19.7% | 20.0% |
| **Comorbid Conditions** |  |  |
| Cancer | 13.1% | 13.1% |
| Cardiovascular disease | 20.3% | 20.3% |
| Chronic heart failure | 18.4% | 18.4% |
| Chronic lung disorders | 19.4% | 20.0% |
| Diabetes | 42.3%* | 43.2% |
| Gastrointestinal disorders | 2.9% | 2.8% |
| HIV | 0.3% | 0.3% |
| Moderate or end-stage liver disease | 1.8% | 1.7% |
| Neurological disorders | 13.5% | 13.7% |
| Pancreatic disease | 1.6% | 1.6% |
| Severe mental health disorders | 6.4% | 6.5% |
| Stroke | 5.8% | 5.9% |
| Substance abuse disorders | 1.9% | 1.9% |
| Severe hematological disorders | 0.5% | 0.5% |
| Vascular disorders | 17.5% | 18.0% |
| **Total number of co-morbid conditions, mean** | **1.7*** | **1.7** |
| *p<0.05 for difference between FQHC users and FQHC Non-Users. | | |

Appendix Table 6: Comparison of utilization between 75,246 Medicare beneficiaries age 65 or older who received primary care at Federally Qualified Health Centers in 2013, and 75,246 propensity-matched Medicare beneficiaries age 65 or older who received primary care elsewhere.

|  | | **FQHC Users**  **(n=75,246)** | | **Matched FQHC Non-Users**  **(n=75,246)** | |  |
| --- | --- | --- | --- | --- | --- | --- |
|  |  | |  |  |  |  |
|  | **Mean number of visits** | | **Percent of benes with at least one** | **Mean number of visits** | **Percent of benes with at**  **least one** |  |
| **Ambulatory visits** |  | |  |  |  |  |
| Any ambulatory visit | 9.6† | | 100.0% | 10.8 | 100.0% |  |
| Primary care | 6.1† | | 100.0% | 6.6 | 100.0% |  |
| Specialty care | 3.5† | | 73.4%† | 5.4 | 78.1% |  |
| At an FQHC | 5.7† | | 100.0%† | 0.0 | 0.0% |  |
| Primary care | 5.5† | | 100.0%† | 0.0 | 0.0% |  |
| Specialty care | 0.2† | | 8.3%† | 0.0 | 0.0% |  |
| At site other than FQHC | 3.9† | | 77.0%† | 10.8 | 100.0% |  |
| Primary care | 0.6† | | 32.2%† | 6.6 | 100.0% |  |
| Specialty care | 3.3† | | 71.0%† | 5.4 | 78.1% |  |
| **ED visits** |  | |  |  |  |  |
| Any ED visit | 0.8† | | 38.6%† | 0.7 | 35.1% |  |
| Chronic ACSC | 0.1† | | 5.4%† | 0.1 | 4.5% |  |
| Other | 0.7† | | 36.7%† | 0.6 | 33.5% |  |
| With admission only | 0.2† | | 12.6%* | 0.2 | 11.9% |  |
| Chronic ACSC | 0.0* | | 2.1%* | 0.0 | 1.9% |  |
| Other | 0.2* | | 11.3%* | 0.1 | 10.8% |  |
| Without admission only | 0.6† | | 33.2%† | 0.5 | 29.4% |  |
| Chronic ACSC | 0.0† | | 3.7%† | 0.0 | 3.0% |  |
| Other | 0.6† | | 31.7%† | 0.5 | 28.1% |  |
| **Hospitalizations** |  | |  |  |  |  |
| Any hospitalization | 0.3† | | 18.9%* | 0.3 | 18.1% |  |
| Chronic ACSC | 0.0* | | 2.8%* | 0.0 | 2.5% |  |
| Other | 0.3† | | 17.4%* | 0.2 | 16.9% |  |
| For comparison with FQHC Non-Users: *p<0.05; †p<0.001; otherwise not statistically significant.  FQHC: Federally-qualified health center; ACSC: Ambulatory Care Sensitive Condition; ED: Emergency Department; E&M: Evaluation and Management. | | | | | | |
|  | | | | | | |

Appendix Table 7: Comparison of baseline characteristics between 130,523 Medicare beneficiaries who received primary care at Federally Qualified Health Centers in 2013, and 130,523 propensity-matched Medicare beneficiaries who received primary care elsewhere, but never visited a Rural Health Clinic.

| **Characteristic** | **FQHC Users**  **(n=130,523)** | **FQHC Non-Users**  **(n=130,523)** |
| --- | --- | --- |
| **Demographics** |  |  |
| Age |  |  |
| 18-64 | 42.3% | 42.5% |
| 65-74 | 36.1% | 35.8% |
| 75-84 | 16.2% | 16.3% |
| >=85 | 5.4% | 5.4% |
| Race/Ethnicity |  |  |
| White | 67.7% | 67.3% |
| Black | 18.8% | 20.2% |
| Hispanic | 7.1% | 6.1% |
| Asian | 2.7% | 2.9% |
| Other/ Unknown | 3.7% | 3.6% |
| Gender |  |  |
| Male | 41.4% | 40.8% |
| Female | 58.6% | 59.2% |
| Disabled | 52.2% | 52.9% |
| End-Stage Renal Disease (ESRD) status | 0.8% | 0.8% |
| Previously Institutionalized | 3.3% | 3.3% |
| Medicaid-eligible | 56.5% | 56.8% |
| Region |  |  |
| Northeast | 19.7% | 18.9% |
| Midwest | 18.1% | 18.6% |
| South | 34.2% | 36.8% |
| West | 28.0% | 25.7% |
| Urban/Rural status, % |  |  |
| Urban | 72.8% | 72.7% |
| Rural/ Isolated | 27.2% | 27.3% |
| **Neighborhood socioeconomic status (SES)** |  |  |
| Median annual household income, mean | $44,569 | $44,356 |
| Percent of residents with less than high school diploma (age 25+), mean | 18.4% | 18.5% |
| Percent of male residents who are unemployed (age 16+), mean | 12.4% | 12.6% |
| Percent of female-headed households with children, mean | 12.8%* | 13.1% |
| Percent of households with public assistance income, mean | 3.8% | 3.8% |
| Percent of individuals with annual income below the FPL, mean | 20.1% | 20.4% |
| **Comorbid Conditions** |  |  |
| Cancer | 10.9% | 10.9% |
| Cardiovascular disease | 16.2% | 16.4% |
| Chronic heart failure | 16.8%* | 17.4% |
| Chronic lung disorders | 19.4%* | 20.0% |
| Diabetes | 40.5%* | 41.2% |
| Gastrointestinal disorders | 3.0% | 3.1% |
| HIV | 1.3% | 1.2% |
| Moderate or end-stage liver disease | 3.2% | 3.1% |
| Neurological disorders | 17.9% | 18.2% |
| Pancreatic disease | 2.0% | 2.0% |
| Severe mental health disorders | 17.5% | 17.5% |
| Stroke | 5.6% | 5.7% |
| Substance abuse disorders | 5.6% | 5.4% |
| Severe hematological disorders | 0.5% | 0.5% |
| Vascular disorders | 14.8%* | 15.2% |
| **Total number of co-morbid conditions, mean** | **1.8*** | **1.8** |
| *p<0.05 for difference between FQHC users and FQHC Non-Users. | | |

Appendix Table 8: Comparison of utilization between 130,523 Medicare beneficiaries who received primary care at Federally Qualified Health Centers in 2013, and 130,523 propensity-matched Medicare beneficiaries who received primary care elsewhere, but never visited a Rural Health Clinic.

|  | | **FQHC Users**  **(n=130,523)** | | **Matched FQHC Non-Users**  **(n=130,523)** | |  |
| --- | --- | --- | --- | --- | --- | --- |
|  |  | |  |  |  |  |
|  | **Mean number of visits** | | **Percent of benes with at least one** | **Mean number of visits** | **Percent of benes with at**  **least one** |  |
| **Ambulatory visits** |  | |  |  |  |  |
| Any ambulatory visit | 10.0† | | 100.0% | 11.3 | 100.0% |  |
| Primary care | 6.2† | | 100.0% | 6.8 | 100.0% |  |
| Specialty care | 3.8† | | 73.7%† | 4.5 | 78.2% |  |
| At an FQHC | 5.8† | | 100.0%† | 0.0 | 0.0% |  |
| Primary care | 5.5† | | 100.0%† | 0.0 | 0.0% |  |
| Specialty care | 0.3† | | 9.8%† | 0.0 | 0.0% |  |
| At site other than FQHC | 4.2† | | 77.3%† | 11.3 | 100.0% |  |
| Primary care | 0.7† | | 33.7%† | 6.8 | 100.0% |  |
| Specialty care | 3.5† | | 70.9%† | 4.5 | 78.2% |  |
| **ED visits** |  | |  |  |  |  |
| Any ED visit | 1.2† | | 44.5%† | 1.0 | 41.1% |  |
| Chronic ACSC | 0.1† | | 5.9%† | 0.1 | 4.8% |  |
| Other | 1.1† | | 42.7%† | 0.9 | 39.7% |  |
| With admission only | 0.2 | | 13.3%* | 0.2 | 12.9% |  |
| Chronic ACSC | 0.0* | | 2.2%* | 0.0 | 2.0% |  |
| Other | 0.2 | | 11.9% | 0.2 | 11.7% |  |
| Without admission only | 1.0† | | 39.8%† | 0.8 | 35.9% |  |
| Chronic ACSC | 0.1† | | 4.3%† | 0.0 | 3.2% |  |
| Other | 0.9† | | 38.3%† | 0.8 | 34.7% |  |
| **Hospitalizations** |  | |  |  |  |  |
| Any hospitalization | 0.3 | | 19.1%* | 0.3 | 18.6% |  |
| Chronic ACSC | 0.0* | | 2.9%† | 0.0 | 2.6% |  |
| Other | 0.3 | | 17.6% | 0.3 | 17.3% |  |
| For comparison with FQHC Non-Users: *p<0.05; †p<0.001; otherwise not statistically significant.  FQHC: Federally-qualified health center; ACSC: Ambulatory Care Sensitive Condition; ED: Emergency Department; E&M: Evaluation and Management. | | | | | | |
|  | | | | | | |

Appendix Table 9: Comparison of baseline characteristics between 96,188 Medicare beneficiaries who received primary care at Federally Qualified Health Centers in 2013, and 96,188 propensity-matched Medicare beneficiaries who received primary care elsewhere. For this sensitivity analysis, the sample was limited to beneficiaries who received at least one visit with a specialist during 2013 (both FQHC users and matched FQHC Non-Users).

| **Characteristic** | **FQHC Users**  **(n=96,188)** | **FQHC Non-Users**  **(n=96,188)** |
| --- | --- | --- |
| **Demographics** |  |  |
| Age |  |  |
| 18-64 | 42.6% | 42.5% |
| 65-74 | 35.4% | 35.3% |
| 75-84 | 16.6% | 16.8% |
| >=85 | 5.4% | 5.4% |
| Race/Ethnicity |  |  |
| White | 68.7% | 68.7% |
| Black | 18.4% | 19.4% |
| Hispanic | 6.9% | 5.9% |
| Asian | 2.4% | 2.6% |
| Other/ Unknown | 3.6% | 3.4% |
| Gender* |  |  |
| Male | 41.1% | 40.5% |
| Female | 58.9% | 59.5% |
| Disabled | 52.8% | 53.4% |
| End-Stage Renal Disease (ESRD) status | 1.0% | 1.0% |
| Previously Institutionalized | 3.9% | 4.0% |
| Medicaid-eligible | 56.8% | 57.2% |
| Region |  |  |
| Northeast | 20.8% | 20.2% |
| Midwest | 18.5% | 19.2% |
| South | 33.9% | 35.7% |
| West | 26.8% | 24.8% |
| Urban/Rural status, % |  |  |
| Urban | 73.3% | 73.0% |
| Rural/ Isolated | 26.7% | 27.0% |
| **Neighborhood socioeconomic status (SES)** |  |  |
| Median annual household income, mean | $44,835 | $44,624 |
| Percent of residents with less than high school diploma (age 25+), mean | 18.2% | 18.3% |
| Percent of male residents who are unemployed (age 16+), mean | 12.3% | 12.5% |
| Percent of female-headed households with children, mean | 12.7% | 13.0% |
| Percent of households with public assistance income, mean | 3.8% | 3.8% |
| Percent of individuals with annual income below the FPL, mean | 19.9% | 20.2% |
| **Comorbid Conditions** |  |  |
| Cancer | 13.5% | 13.4% |
| Cardiovascular disease | 19.6% | 19.8% |
| Chronic heart failure | 19.8% | 20.1% |
| Chronic lung disorders | 21.4% | 21.9% |
| Diabetes | 42.6%* | 43.7% |
| Gastrointestinal disorders | 3.7% | 3.7% |
| HIV | 1.3% | 1.2% |
| Moderate or end-stage liver disease | 3.7% | 3.5% |
| Neurological disorders | 20.6% | 21.0% |
| Pancreatic disease | 2.4% | 2.4% |
| Severe mental health disorders | 20.1% | 20.1% |
| Stroke | 6.2% | 6.3% |
| Substance abuse disorders | 6.2%* | 5.8% |
| Severe hematological disorders | 0.6% | 0.5% |
| Vascular disorders | 17.9%* | 18.7% |
| **Total number of co-morbid conditions, mean** | 2.0* | 2.0 |
| *p<0.05 for difference between FQHC users and FQHC Non-Users. | | |

Appendix Table 10: Comparison of utilization between 96,188 Medicare beneficiaries who received primary care at Federally Qualified Health Centers in 2013, and 96,188 propensity-matched Medicare beneficiaries who received primary care elsewhere. For this sensitivity analysis, the sample was limited to beneficiaries who received at least one visit with a specialist during 2013 (both FQHC users and matched FQHC Non-Users).

|  | | **FQHC Users**  **(n=96,188)** | | **Matched FQHC Non-Users**  **(n=96,188)** | |  |
| --- | --- | --- | --- | --- | --- | --- |
|  |  | |  |  |  |  |
|  | **Mean number of visits** | | **Percent of benes with at least one** | **Mean number of visits** | **Percent of benes with at**  **least one** |  |
| **Ambulatory visits** |  | |  |  |  |  |
| Any ambulatory visit | 11.7† | | 100.0% | 13.0 | 100.0% |  |
| Primary care | 6.6† | | 100.0% | 7.1 | 100.0% |  |
| Specialty care | 5.2† | | 100.0% | 5.8 | 100.0% |  |
| At an FQHC | 6.2† | | 100.0%† | 0.0 | 0.0% |  |
| Primary care | 5.8† | | 100.0%† | 0.0 | 0.0% |  |
| Specialty care | 0.4† | | 13.3%† | 0.0 | 0.0% |  |
| At site other than FQHC | 5.6† | | 97.0%† | 13.0 | 100.0% |  |
| Primary care | 0.8† | | 37.8%† | 7.1 | 100.0% |  |
| Specialty care | 4.8† | | 96.3%† | 5.8 | 100.0% |  |
| **ED visits** |  | |  |  |  |  |
| Any ED visit | 1.3† | | 48.8%† | 1.1 | 45.3% |  |
| Chronic ACSC | 0.1† | | 6.6%† | 0.1 | 5.6% |  |
| Other | 1.2† | | 47.1%† | 1.1 | 43.7% |  |
| With admission only | 0.2* | | 15.5%* | 0.2 | 14.8% |  |
| Chronic ACSC | 0.0* | | 2.6%* | 0.0 | 2.4% |  |
| Other | 0.2* | | 14.0%* | 0.2 | 13.5% |  |
| Without admission only | 1.1† | | 43.6%† | 0.9 | 39.6% |  |
| Chronic ACSC | 0.1† | | 4.7%† | 0.1 | 3.7% |  |
| Other | 1.0† | | 42.1%† | 0.9 | 38.3% |  |
| **Hospitalizations** |  | |  |  |  |  |
| Any hospitalization | 0.4 | | 22.5%* | 0.4 | 22.0% |  |
| Chronic ACSC | 0.0 | | 3.3%* | 0.0 | 3.1% |  |
| Other | 0.3 | | 20.9% | 0.3 | 20.5% |  |
| For comparison with FQHC Non-Users: *p<0.05; †p<0.001; otherwise not statistically significant.  FQHC: Federally-qualified health center; ACSC: Ambulatory Care Sensitive Condition; ED: Emergency Department; E&M: Evaluation and Management. | | | | | | |
|  | | | | | | |

Appendix Table 11: Comparison of baseline characteristics between 61,908 Medicare beneficiaries who received primary care at Federally Qualified Health Centers in 2013, and 61,908 propensity-matched Medicare beneficiaries who received primary care elsewhere. For this sensitivity analysis, the sample was limited to beneficiaries who received at least three visits with a specialist during 2013 (both FQHC users and matched FQHC Non-Users).

| **Characteristic** | **FQHC Users**  **(n=61,908)** | **FQHC Non-Users**  **(n=61,908)** |
| --- | --- | --- |
| **Demographics** |  |  |
| Age |  |  |
| 18-64 | 44.5% | 44.6% |
| 65-74 | 33.7% | 33.8% |
| 75-84 | 16.6% | 16.4% |
| >=85 | 5.2% | 5.1% |
| Race/Ethnicity |  |  |
| White | 69.4% | 69.5% |
| Black | 18.4% | 19.0% |
| Hispanic | 6.7% | 6.0% |
| Asian | 2.2% | 2.3% |
| Other/ Unknown | 3.4% | 3.2% |
| Gender |  |  |
| Male | 40.8% | 40.6% |
| Female | 59.2% | 59.4% |
| Disabled | 55.3% | 55.8% |
| End-Stage Renal Disease (ESRD) status | 1.2% | 1.3% |
| Previously Institutionalized | 4.4% | 4.5% |
| Medicaid-eligible | 58.3% | 58.3% |
| Region |  |  |
| Northeast | 21.7% | 21.5% |
| Midwest | 18.5% | 19.1% |
| South | 34.2% | 35.4% |
| West | 25.5% | 24.1% |
| Urban/Rural status, % |  |  |
| Urban | 74.6% | 74.7% |
| Rural/ Isolated | 25.4% | 25.3% |
| **Neighborhood socioeconomic status (SES)** |  |  |
| Median annual household income, mean | $45,085 | $44,898 |
| Percent of residents with less than high school diploma (age 25+), mean | 18.2% | 18.2% |
| Percent of male residents who are unemployed (age 16+), mean | 12.3% | 12.5% |
| Percent of female-headed households with children, mean | 12.8% | 13.1% |
| Percent of households with public assistance income, mean | 3.8% | 3.8% |
| Percent of individuals with annual income below the FPL, mean | 19.9% | 20.1% |
| **Comorbid Conditions** |  |  |
| Cancer | 16.5% | 16.5% |
| Cardiovascular disease | 22.8% | 23.1% |
| Chronic heart failure | 23.2% | 23.7% |
| Chronic lung disorders | 24.2% | 24.6% |
| Diabetes | 44.6% | 45.2% |
| Gastrointestinal disorders | 4.4% | 4.6% |
| HIV | 1.4% | 1.3% |
| Moderate or end-stage liver disease | 4.2% | 4.1% |
| Neurological disorders | 23.8% | 23.9% |
| Pancreatic disease | 2.8% | 2.9% |
| Severe mental health disorders | 24.1% | 24.2% |
| Stroke | 6.8% | 6.9% |
| Substance abuse disorders | 7.1% | 7.0% |
| Severe hematological disorders | 0.7% | 0.7% |
| Vascular disorders | 21.4% | 21.7% |
| **Total number of co-morbid conditions, mean** | **2.3** | **2.3** |
| *p<0.05 for difference between FQHC users and FQHC Non-Users. | | |

Appendix Table 12: Comparison of utilization between 61,908 Medicare beneficiaries who received primary care at Federally Qualified Health Centers in 2013, and 61,908 propensity-matched Medicare beneficiaries who received primary care elsewhere. For this sensitivity analysis, the sample was limited to beneficiaries who received at least three visits with a specialist during 2013 (both FQHC users and matched FQHC Non-Users).

|  | | **FQHC Users**  **(n=61,908)** | | **Matched FQHC Non-Users**  **(n=61,908)** | |  |
| --- | --- | --- | --- | --- | --- | --- |
|  |  | |  |  |  |  |
|  | **Mean number of visits** | | **Percent of benes with at least one** | **Mean number of visits** | **Percent of benes with at**  **least one** |  |
| **Ambulatory visits** |  | |  |  |  |  |
| Any ambulatory visit | 14.2† | | 100.0% | 15.3 | 100.0% |  |
| Primary care | 7.0† | | 100.0% | 7.5 | 100.0% |  |
| Specialty care | 7.2† | | 100.0% | 7.9 | 100.0% |  |
| At an FQHC | 6.6† | | 100.0%† | 0.0 | 0.0% |  |
| Primary care | 6.1† | | 100.0%† | 0.0 | 0.0% |  |
| Specialty care | 0.5† | | 15.7%† | 0.0 | 0.0% |  |
| At site other than FQHC | 7.6† | | 98.3%† | 15.3 | 100.0% |  |
| Primary care | 0.9† | | 41.3%† | 7.5 | 100.0% |  |
| Specialty care | 6.7† | | 97.9%† | 7.9 | 100.0% |  |
| **ED visits** |  | |  |  |  |  |
| Any ED visit | 1.5† | | 53.6%† | 1.3 | 49.4% |  |
| Chronic ACSC | 0.1† | | 7.7%† | 0.1 | 6.6% |  |
| Other | 1.4† | | 51.7%† | 1.2 | 47.7% |  |
| With admission only | 0.3 | | 18.2%* | 0.3 | 17.2% |  |
| Chronic ACSC | 0.0* | | 3.2%* | 0.0 | 2.9% |  |
| Other | 0.2 | | 16.4%* | 0.2 | 15.7% |  |
| Without admission only | 1.2† | | 47.7%† | 1.1 | 43.1% |  |
| Chronic ACSC | 0.1† | | 5.4%† | 0.1 | 4.3% |  |
| Other | 1.2† | | 46.2%† | 1.0 | 41.7% |  |
| **Hospitalizations** |  | |  |  |  |  |
| Any hospitalization | 0.4 | | 26.5%* | 0.4 | 25.5% |  |
| Chronic ACSC | 0.1* | | 4.1%* | 0.1 | 3.7% |  |
| Other | 0.4 | | 24.6%* | 0.4 | 23.9% |  |
| For comparison with FQHC Non-Users: *p<0.05; †p<0.001; otherwise not statistically significant.  FQHC: Federally-qualified health center; ACSC: Ambulatory Care Sensitive Condition; ED: Emergency Department; E&M: Evaluation and Management. | | | | | | |
|  | | | | | | |

Appendix Table 13: Comparison of utilization between 130,637 Medicare beneficiaries who received primary care at Federally Qualified Health Centers in 2013, and 1,000,000 randomly selected Medicare beneficiaries who received primary care elsewhere. This analysis is controlled for age and health status using a multivariate model. Health status is measured by the thirteen conditions listed in the HCC, the overall HCC score, and the presence of ESRD status.

|  | | **FQHC Users**  **(n=130,637)** | | **Matched FQHC Non-Users**  **(n=1,000,000)** | |  |
| --- | --- | --- | --- | --- | --- | --- |
|  |  | |  |  |  |  |
|  | **Mean number of visits** | | **Percent of benes with at least one** | **Mean number of visits** | **Percent of benes with at**  **least one** |  |
| **Ambulatory visits** |  | |  |  |  |  |
| Any ambulatory visit | 10.4† | | 100.0% | 11.9 | 100.0% |  |
| Primary care | 6.0† | | 100.0% | 6.3 | 100.0% |  |
| Specialty care | 4.3† | | 78.1%† | 5.5 | 85.9%% |  |
| At an FQHC | 5.8† | | 100.0%† | 0.0 | 0.0% |  |
| Primary care | 5.5† | | 100.0%† | 0.0 | 0.0% |  |
| Specialty care | 0.3† | | 9.6%† | 0.0 | 0.1% |  |
| At site other than FQHC | 4.6† | | 78.1%† | 11.9 | 100.0% |  |
| Primary care | 0.5† | | 33.8%† | 6.4 | 100.0% |  |
| Specialty care | 4.1† | | 75.5%† | 5.5 | 86.0% |  |
| **ED visits** |  | |  |  |  |  |
| Any ED visit | 1.6† | | 51.6%† | 1.4 | 46.3% |  |
| Chronic ACSC | 0.1† | | 8.6%† | 0.1 | 7.2% |  |
| Other | 1.5† | | 50.2%† | 1.3 | 45.2% |  |
| With admission only | 0.5 | | 24.0% | 0.5 | 23.8% |  |
| Chronic ACSC | 0.1† | | 4.4%† | 0.1 | 4.1% |  |
| Other | 0.4 | | 22.5% | 0.4 | 22.5% |  |
| Without admission only | 1.2† | | 44.8%† | 0.9 | 38.6% |  |
| Chronic ACSC | 0.1† | | 5.4%† | 0.1 | 4.0% |  |
| Other | 1.1† | | 43.5%† | 0.9 | 37.6% |  |
| **Hospitalizations** |  | |  |  |  |  |
| Any hospitalization | 0.7* | | 30.8%* | 0.7 | 31.2% |  |
| Chronic ACSC | 0.1† | | 6.0%† | 0.1 | 5.7% |  |
| Other | 0.6† | | 29.1%† | 0.6 | 29.7% |  |
| For comparison with FQHC Non-Users: *p<0.05; †p<0.001; otherwise not statistically significant.  FQHC: Federally-qualified health center; ACSC: Ambulatory Care Sensitive Condition; ED: Emergency Department; E&M: Evaluation and Management. | | | | | | |
|  | | | | | | |
